# Imported *Mansonella* perstans infection in Spain

**DOI:** 10.1186/s40249-020-00729-9

**Published:** 2020-07-23

**Authors:** Sabino Puente, Mar Lago, Mercedes Subirats, Ismael Sanz-Esteban, Marta Arsuaga, Belén Vicente, Montserrat Alonso-Sardon, Moncef Belhassen-Garcia, Antonio Muro

**Affiliations:** 1 Unidad de Medicina Tropical, Servicio de Medicina Interna, Hospital La Paz-Carlos III, Madrid, Spain; 2 Microbiología, Hospital La Paz-Carlos III, Madrid, Spain; 3 Facultad de Fisioterapia, Universidad Europea de Madrid, Madrid, Spain; 4 Laboratorio de Inmunología Parasitaria y Molecular, CIETUS, IBSAL, Facultad de Farmacia, Universidad de Salamanca, 37007 Salamanca, Spain; 5 Área de Medicina Preventiva y Salud Pública, IBSAL, CIETUS, Universidad de Salamanca, Salamanca, Spain; 6 Servicio de Medicina Interna, Sección de Enfermedades Infecciosas, CAUSA, IBSAL, CIETUS, Universidad de Salamanca, Paseo San Vicente 58-182, 37007 Salamanca, Spain

**Keywords:** Mansonellosis, *Mansonella perstans*, Clinical study, Immigrant, Imported diseases, Spain

## Abstract

**Background:**

*Mansonella perstans* infection can be considered one of the most neglected tropical infectious diseases. Very few studies have reported on the clinical picture caused by infection with this nematode. Therefore, our study was aimed to describe the clinical patterns and treatment of imported *M. perstans* infection by migrants from Africa.

**Methods:**

The present study evaluated a large cohort of migrants who have been diagnosed, examined and treated for imported *M. perstans* infection at a Spanish reference center (Hospital Carlos III Tropical Medicine Unit, Madrid, Spain) over a 19-year period. Most patients voluntarily attend the emergency unit or are referred from primary care or general hospitals in Madrid. Chi-square test was used to compare the association between categorical variables. The continuous variables were compared by Student’s *t-*test or the Mann–Whitney test. The corresponding regression models were used for multivariate analysis.

**Results:**

Five hundred three cases of migrants from tropical and subtropical areas with *M. perstans* infection were identified. Two hundred sixty-four patients were female (52.5%). The mean age (± SD) was 44.6 ± 18.2 years (range: 16–93 years). The mean time (± SD) between the arrival in Spain and the first consultation was 8.6 ± 18.0 months. The major origin of the patients was Equatorial Guinea (97.6%). Regarding the clinical picture, 257 patients were asymptomatic (54.7%) and 228 were symptomatic (45.3%); 190 patients had pruritus (37.8%), 50 (9.9%) had arthralgia, 18 patients had Calabar-like swelling (3.6%), and 15 (3%) had abdominal pain. Four hundred forty-two (87.9%) migrants had hyper-IgE, and 340 (67.6%) had eosinophilia. One hundred ninety-five patients had coinfections with other filarial nematodes (38.8%), and 308 migrants had only *M. perstans* infection (61.2%). Four hundred thirty-seven cases (86.9%) had been treated with anti-filarial drugs; 292 cases were treated with one anti-filarial drug, and 145 cases were treated with combined anti-filarial therapy. Additionally, 20 (4%) cases received steroids and 38 (7.6%) cases received antihistamines.

**Conclusions:**

A long series of *M. perstans* infections is presented in sub-Saharan immigrants whose data indicate that it should be included in the differential diagnosis in patients with pruritus or analytical alterations such as eosinophilia or hyper-IgE presentation, and they also have a high number of coinfections with other microorganisms whose treatment needs to be protocolized.

## Introduction

*Mansonella perstans* is transmitted by biting midges (*Culicoides*). The life cycle is similar to that of other filariae. Microfilariae are responsible for the transmission of infection because they are taken up during the blood meal of the insect vector. The epidemiology of *M. perstans* has not been clearly defined. Among the known human filarial infections, mansonellosis is probably the most frequent filariasis in sub-Saharan Africa as well as a northern part of the Amazon rainforest stretching from equatorial Brazil to the Caribbean coast of South America [1]. It has been estimated that 114 million people may be infected and as many as 581 million people in 33 countries are at risk for *M. perstans* infection in Africa alone [2]. Many publications refer to mansonellosis as one of the most common human helminthiases in endemic areas, and it is more prevalent and more neglected than other filarial diseases such as lymphatic filariasis, onchocerciasis, and loiasis. In endemic areas, the probability of infection increases with age, with the prevalence reaching 100% in highly endemic areas. However, the infection is the least studied and is likely one of the most neglected of all tropical diseases, subject to more neglect than schistosomiasis, taeniasis, echinococcosis, or rabies [1, 3].

The adult parasites are thought to live in serous body cavities, and the female parasites release microfilariae into the blood [4]. The diagnosis of *M. perstans* infection is usually by detection and identification of the microfilariae that circulate in the blood. Because the microfilariae are present in the peripheral blood in almost equal concentrations during the day and night [5], blood samples for the diagnosis can be obtained at any time. The prevalence and intensity of microfilaremia increase gradually with age. Few and old studies have reported on the clinical picture caused by infection with *M. perstans* nematodes because the parasite is widespread in remote areas [2, 6]. Usually, infected people have other parasitic infections that could contribute to clinical manifestations [2, 6]. Clinical manifestations seem to be related to adult parasites than to the microfilariae, and the symptoms are probably related to the migration of the worms, including transient subcutaneous swellings (similar to the Calabar swellings caused by *Loa loa*), pruritus, rash, urticaria, arthralgia, abdominal pain, eosinophilia, fatigue, pericarditis, pleuritis and inflammatory granulomatous nodules surrounding dead adult worms [2]. Currently, no standard treatment exists for mansonellosis and its handling is still debatable. Therefore, many drugs have been used alone or combined, such as diethylcarbamazine, ivermectin, mebendazole, levamisole, albendazole, doxycycline and thiabendazole [1, 2, 7].

Despite accumulating evidence of a high prevalence of human infections, no current large-scale filariasis control program has targeted mansonellosis. Mansonellosis is not listed among the neglected diseases of the World Health Organization, and no control strategy has been defined against this human filariasis.

The health-related impact on individuals living with these filariae remains unknown, and evidence regarding treatment strategies is scarce. Like other neglected diseases, it mainly affects poor populations living in tropical and subtropical climates and it has not been associated with a clear and distinct clinical picture [2].

In summary, the health-related impact on people living with these filariae remains unknown, and it can be considered one of the most neglected tropical infectious diseases [3]. Therefore, our study was aimed to describe the clinical patterns and treatment of imported *M. perstans* infection by migrants from Africa.

## Material and methods

### Study

The La Paz-Carlos III Hospital in Madrid, Spain, is a tropical disease referral unit. Most patients voluntarily attend the emergency unit or are referred from primary care or general hospitals in Madrid. A very small percentage of patients come from other regions.

A retrospective study was conducted on the data regarding immigrants diagnosed with *M. perstans* infection over a 19-year period. The diagnosis of *M. perstans* infections was established with confirmed microfilaremia. The direct detection of circulating microfilaria was performed on fresh venous blood obtained around midday with a thick film and/or thin smear after Giemsa staining. Microfilaremia was occasionally estimated on thin smears. The exclusion criteria included diagnosis in travelers, unspecified diagnosis methods (i.e., clinical data only), and medical records with missing data. The data included demographics (age, gender, nationality, time of the first consultation) and clinical characteristics (symptoms and when the symptoms first appeared). The eye examination results and analytical data regarding serologic tests for syphilis, HIV, hepatitis B and C, eosinophil counts, IgE levels and stool test results regarding ova and parasites were reviewed. Other laboratory test results were also recorded. Systematic ophthalmology exploration was performed in patients with a clinical suspicion of onchocerciasis. Hyper-IgE was defined as an increase in peripheral blood IgE to more than 200 U/ml. Hyper-IgE was classified as being mild (> 200–399 U/ml), moderate (> 399–999 U/ml) and/or severe (> 1000 U/ml). Relative eosinophilia was defined as an elevated percentage of eosinophils (> 5%) in individuals with < 450 ×  10^6^ eosinophils/L. Absolute eosinophilia was defined as an increase in the peripheral blood eosinophilic leukocytes to more than 450 × 10^6^ eosinophils/L of blood. Mild eosinophilia was defined as > 450 × 10^6^ eosinophils/L to 999 × 10^6^ eosinophils/L. Moderate eosinophilia was defined as > 1000 × 10^6^ eosinophils/L to 2999 × 10^6^ eosinophils/L, and severe eosinophilia was defined as > 3000 × 10^6^ eosinophils/L.

### Statistical analysis

Categorical variable results were expressed as percentages and as the mean and standard deviation (SD) for continuous variables. Chi-square test was used to compare the association between the categorical variables (i.e., clinical and demographic variables). The measured outcomes were expressed as the odds ratio (*OR*) with a 95% confidence interval (*CI*). The continuous variables were compared by Student’s *t*-test or the Mann–Whitney test for two groups depending on their normal or non-normal distribution. The corresponding regression models were used for multivariate analysis considering *P* < 0.05 for a statistically significant difference. The Statistical Package for the Social Sciences (SPSS 23.0®; IBM Corp., Armonk, New York, USA) was used to analyze all the data.

## Results

### Demographic and epidemiological data

In total, 503 cases of *M. perstans* infection were identified at the Carlos III Hospital. The main epidemiological data are shown in Table 1**.** Two hundred sixty-four patients were female (52.5%). The migrants’ mean age (±SD) was 44.6 ± 18.2 years (range: 16–93); the median (25th, 75th percentiles) age was 43 years (28, 60). Most of them (97.6%) came from Equatorial Guinea, 12 cases came from other African countries: D.R.Congo (3), Cameroon (2), Guinea Bissau (2), Nigeria (2), Gabon (1), Guinea Conakry (1) and Togo (1). All the patients were infected in their country of origin. The mean time (± SD) between their arrival to Spain and their first medical consultation was 8.6 ± 18.0 months (range: 1–180); the median (25th, 75th percentiles) time was 2 months (1, 7). Half of the patients (50.3%) were evaluated during the first month of stay in Spain. The mean number of cases (± SD) was 25.1 ± 11.8 (5–45) per month, with an irregular chronological distribution.

**Table 1:** Epidemiological, clinical, laboratory and evolution data, according to microbiological (*Mansonella perstans* vs co-infections) and clinical associations (symptomatic vs asymptomatic)

	All patients	Microbiological association			Clinical association		
	*N* = 503 (100%)	Only M. perstans *n*_1_ = 308 (61.2%)	Co-infections *n*_2_ = 195 (38.8%)	*P*-value*	Symptomatic *n*_3_ = 228 (45.3%)	Asymptomatic *n*_4_ = 275 (54.7%)	*P*-value*
**Epidemiological data**							
Male	239 (47.5)	149 (48.4)	90 (46.2)	0.627	94 (41.2)	145 (52.7)	0.010*
Female	264 (52.5)	159 (51.6)	105 (53.8)		134 (58.8)	130 (47.3)	
Age, mean ± SD, years	44.6 ± 18.2	45.4 ± 17.8	43.3 ± 18.7	0.228	44.8 ± 18.6	44.4 ± 17.8	0.771
0–24	93 (18.5)	50 (16.2)	43 (22.1)		44 (19.3)	49 (17.8)	
25–50	205 (40.8)	129 (41.9)	76 (39.0)		93 (40.8)	112 (40.7)	
> 50	205 (40.8)	129 (41.9)	76 (39.0)		91 (39.9)	114 (41.5)	
Months in Spain, mean ± SD	8.6 ± 18.0	8.2 ± 19.9	9.2 ± 14.4	0.540	8.4 ± 15.7	8.7 ± 19.7	0.850
**Clinical data**							
Asymptomatic	275 (54.7)	194 (63.0)	81 (41.5)	< 0.001*			
Symptomatic	228 (45.3)	114 (37.0)	114 (58.5)				
Pruritus	190 (37.8)	88 (28.6)	102 (52.3)				
Arthralgia	50 (9.9)	33 (10.7)	17 (8.7)				
Abdominal pain	15 (3.0)	12 (3.9)	3 (1.5)				
Subcutaneous step (calabar swelling)	18 (3.6)	3 (1.0)	15 (7.7)				
**Laboratory data**							
Eosinophilia, × 10^6^ eosinophils/L	*N* = 503	*n*_1_ = 308	*n*_2_ = 195		*n*_3_ = 228	*n*_4_ = 275	
Without eosinophilia (< 450)	105 (20.9)	69 (22.4)	36 (18.5)	< 0.001*	54 (23.7)	51 (18.5)	< 0.001*
Relative eosinophilia (< 450 + > 5%)	58 (11.5)	54 (17.5)	4 (2.1)		22 (9.6)	36 (13.1)	
Mild eosinophilia (450–999)	159 (31.6)	104 (33.8)	55 (28.2)		53 (23.2)	106 (38.5)	
Moderate eosinophilia (1000–2999)	154 (30.6)	75 (24.4)	79 (40.5)		78 (34.2)	76 (27.6)	
Severe eosinophilia (≥ 3000)	27 (5.4)	6 (1.9)	21 (10.8)		21 (9.2)	6 (2.2)	
Mean ± SD	1151.7 ± 1296.5	819.8 ± 863.0	1465.4 ± 1707.2		1252.0 ± 1387.7	919.2 ± 1197.4	
Immunoglobulin E, U/ml	*N* = 491	*n*_1_ = 300	*n*_2_ = 191		*n*_3_ = 222	*n*_4_ = 269	
Normal (< 200)	49 (10.0)	37 (12.3)	12 (6.3)	0.001*	20 (9.0)	29 (10.8)	0.749
Mild hyper-IgE (200–399)	57 (11.6)	46 (15.3)	11 (5.8)		23 (10.4)	34 (12.6)	
Moderate hyper-IgE (400–999)	105 (21.4)	61 (20.3)	44 (23.0)		48 (21.6)	57 (21.2)	
Severe hyper-IgE (≥ 1000)	280 (55.7)	156 (52.0)	124 (64.9)		131 (59.0)	149 (55.4)	
Mean ± SD	1417.3 ± 1132.9	1310.0 ± 1111.7	1585.8 ± 1148.2		1442.2 ± 1153.7	1396.8 ± 1117.2	
**Evolution**							
Healing**	240 (47.7)	147 (47.7)	93 (47.7)	0.599	118 (51.8)	122 (44.4)	0.247
No	18 (3.6)	9 (2.9)	9 (4.6)		7 (3.1)	11 (4.0)	
No follow-up	245 (48.7)	152 (49.4)	93 (47.7)		103 (45.2)	142 (51.6)	

*Statistical significance level of 5% (*P* < 0.05). ** Healing was assessed with after negative microfilaremia

### Clinical and laboratory data

The main clinical and analytical patient data are described in Table 1, according to the variables “Microbiological association”, *Mansonella perstans* (*n*_1_ = 308, 61.2%) vs coinfections (*n*_2_ = 195, 38.8%) and “Clinical association”, symptomatic (*n*_3_ = 228, 45.3%) vs asymptomatic (*n*_4_ = 275, 54.7%). Clinical manifestations were observed in 228 patients (45.3%). The most frequent symptom was pruritus in 190 patients (83.3%), arthralgia in 50 patients (9.9%), abdominal pain in 15 patients (3.0%) and Calabar swelling in 18 patients (3.6%). Significant differences (*P* = 0.010) were found between the percentage of males with symptoms (41.2%) and females with symptoms (58.8%), and no significant differences in age were found (*P* = 0.771).

Regarding eosinophilia levels, 105 (20.9%) patients had a normal eosinophil count, 58 (11.5%) had relative eosinophilia and 340 (67.6%) patients had absolute eosinophilia: mild eosinophilia in 159 patients (31.6%), moderate eosinophilia in 154 patients (30.6%) and severe eosinophilia in 27 patients (5.4%). Moreover, 152/228 (66.6%) symptomatic patients had absolute eosinophilia vs 188/275 (68.3%) asymptomatic patients. Statistically significant differences were found between the percentages of symptomatic vs asymptomatic cases and levels of eosinophilia (*P* < 0.001). Concerning IgE detection, 49 (10%) patients had normal levels, and the remainder (442, 90%) had hyper-IgE: mild hyper-IgE (57, 11.6%), moderate hyper-IgE (105, 21.4%) and severe hyper-IgE (280, 55.7%). No statistically significant differences were found between asymptomatic and symptomatic patients and IgE (*P* = 0.749). Finally, 240 (47.7%) cases healed. Among the remaining 263 (52.3%) cases, 245 (48.7%) individuals had no follow-up data and 18 (3.6%) cases were not cured.

### Microbiology data

The microorganism coinfection in patients with *Mansonella perstans* infection showed in Table 2. Globally, 308 migrants (61.2%) had only *M. perstans* infections and 195 patients (38.8%) had coinfection with other filarial nematodes. *Onchocerca volvulus* was the most frequent filarial coinfection with 138 patients (27.4%). One hundred eighty-six patients (37%) had coinfection with other helminths. *Trichuris trichiura* was the most frequent helminthic coinfection, with 137 patients (27.2%). Moreover, 73 (14.5%) patients presented simultaneously with other filarial and helminthic coinfections. Additionally, 78 patients (15.5%) were protozoa coinfected, mainly with amoebas (58, 11.5%). Finally, 26 patients (5.2%) had HIV coinfection.

**Table 2:** Co-infections in patients with *Mansonella perstans* infections

	Total = 503 (100%) *n* (%)
Only *Mansonella perstans*	308 (61.2)
**Filarial co-infection**^**a**^	195 (38.8)
*Onchocerca volvulus*	110 (21.9)
*Loa loa*	48 (9.5)
*Mansonella streptocerca*	7 (1.4)
*Loa loa* + *Onchocerca volvulus*	12 (2.4)
*Onchocerca volvulus* + *Mansonella streptocerca*	13 (2.6)
*Loa loa* + *Onchocerca volvulus* + *Mansonella streptocerca*	3 (0.6)
*Loa loa* + *Mansonella streptocerca* + *Wuchereria bancrofti*	1 (0.2)
All negative^b^	1 (0.2)
**Other helminthic co-infections**^**c**^	186 (37.0)
*Trichuris trichiura*	74 (14.7)
*Ascaris lumbricoides*	33 (6.6)
Hookworms	6 (1.2)
*Strongyloides stercoralis*	5 (1.0)
*Schistosoma* spp.	2 (0.4)
*Trichuris trichiura* + *Ascaris lumbricoides*	44 (8.7)
*Trichuris trichiura* + Hookworms	8 (1.6)
*Trichuris trichiura* + *Schistosoma* spp.	1 (0.2)
*Ascaris lumbricoides* + Hookworms	2 (0.4)
*Ascaris lumbricoides* + *Strongyloides stercoralis*	1 (0.2)
*Trichuris trichiura* + *Ascaris lumbricoides* + Hookworms	9 (1.8)
*Trichuris trichiura* + *Ascaris lumbricoides* + *Strongyloides stercoralis*	1 (0.2)
*M. pertans* + Other filarials + Other helminthics co-infection	73 (14.5)
Protozoa co-infection^c^	78 (15.5)
Amebas	54 (10.7)
*Dientamoeba fragilis*	3 (0.6)
*Giardia lamblia*	17 (3.4)
Amebas + *Giardia lamblia*	4 (0.8)
**Viruses co-infection**	26 (5.2)
HIV	26 (5.2)

^a^ Microfilaremia search ^b^All negative but presence of calabar swelling. ^c^ Parasitological examinations

The presence of coinfections was not significantly related to gender (48.4% males vs 51.6% females, *P* = 0.627). No significant differences were found between age groups (*P* = 0.228). By contrast, a higher percentage of patients infected only with *M. pertans* was asymptomatic (63%) than patients coinfected (58.5%) (*P* < 0.001). Coinfected patients had higher absolute eosinophilia percentages (*P* <  0.001), severe eosinophilia (10.8% vs 1.9%) and moderate eosinophilia (40.5% vs 24.4%). Similarly, coinfected patients had higher hyper-IgE, severe and moderate (*P* = 0.001) (see Table 1).

### Treatment and outcome

**Fig. 1:**
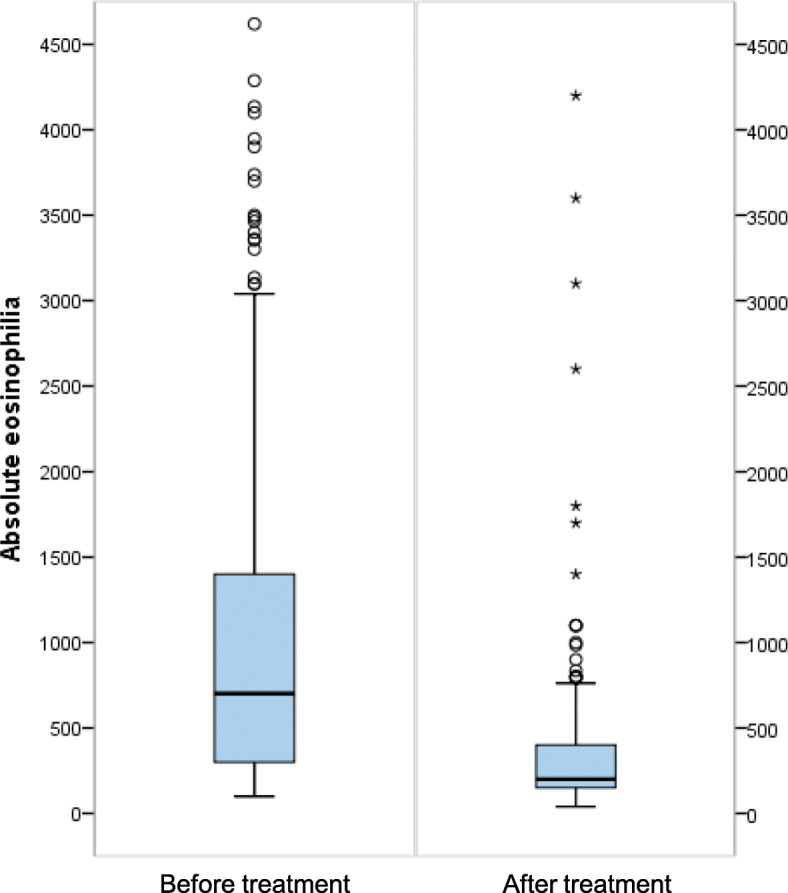
Eosinophilia before and after treatment

Four hundred thirty-seven cases (86.9%) were treated, 66 (13.1%) cases were untreated, and 292 cases (58.1%) used only one drug: mebendazole 100 mg/12 h/30 days (*n* = 267), ivermectin 200 μg/kg single dose (*n* = 16) and albendazole 400 mg/12 h/3 weeks (*n* = 9). By contrast, 145 cases (28.8%) used combined therapy, mainly ivermectin and mebendazole (*n* = 113), as shown in Table 3. Therefore, the drug most used, alone or associated, was mebendazole, in 407 patients. Most of them (*n* = 382) received a single course, 24 double courses and 1 triple courses. Corticosteroid therapy was administered concurrently with the anti-filarial drug in 20 (4%) cases, and an antihistaminic drug was administered with the anti-filarial drug in 38 (7.6%) cases. Figure 1 shows a significant decrease in eosinophilia before and after treatment (*P* < 0.001).

**Table 3:** Treatment in patients with *Mansonella perstans, n/n (proportion, %)*

	Anti-filarial drugs 437/503 (86.9%)	Adverse effects 25/503 (5%)	Healing^a^ 240/503 (47.7%)
**Simple treatment**	292/503 (58.1)	12/292 (4.1)	154/292 (52.7)
Mebendazole	267/503 (53.1)	9/267 (3.4)	152/267 (56.9)
Ivermectine	16/503 (3.2)	2/16 (12.5)	0
Albendazole	9/503 (1.8)	1/9 (11.1)	2/9 (22.2)
**Combined treatment**	145/503 (28.8)	13/145 (9.0)	86/145 (59.3)
Diethylcarbamazcine + Ivermectine + Mebendazole	20/503 (4.0)	3/20 (15.0)	17/20 (85.0)
Diethylcarbamazcine + Mebendazole	3/503 (0.6)	1/3 (33.3)	3/3 (100.0)
Diethylcarbamazcine + Albendazole	5/503 (1.0)	2/5 (40.0)	4/5 (80.0)
Ivermectine + Mebendazole	113/503 (22.5)	7/113 (6.2)	61/113 (54.0)
Ivermectine + Mebendazole + Albendazole	1/503 (0.2)	0	0
Mebendazole + Albendazole	3/503 (0.6)	0	1/3 (33.3)
**None/No follow-up**	66/503 (13.1)	478/503 (95.0)	263/503 (52.3)

^a^ Healing was assessed with after negative microfilaremia and/or remission of Calabar swelling

Adverse reactions to the anti-filarial drugs occurred in 25 (5%) migrants: 15 had pruritus (13 associated with mebendazole and 2 with ivermectin), 3 had pruritus and skin abscess (3 mebendazole), 1 had arthritis (dietilcarbamazine and ivermectin and mebendazole), and 1 had hepatotoxicity (diethylcarbamazine [DEC] and albendazole).

## Discussion

Most symptoms ascribed to *M. perstans* infections in modern scientific literature are based on symptoms that have been recorded in case study reports. Most of these reports have been based on the treatment of tourists and expatriate Europeans and North Americans returning home from endemic areas, and not on people who have lived all their lives in endemic areas [8]. It is unclear whether the symptoms reported from these studies can be used to compile a clinical picture that represents all or even most infections caused by mansonellosis. The identification of these infections is often complicated by coinfection with other infective agents [9]. Bassene et al. analyzed patients infected only with *M. perstans* and concluded that these infections had low pathogenicity because most individuals with *M. perstans* infection are asymptomatic [10, 11]. When symptoms occur, they are predominantly related to the migration of the adult worms and include dermatological symptoms such as transient subcutaneous swellings similar to the Calabar swellings of *Loa loa* infection, serositis (i.e., pericarditis and pleuritis) [12, 13], and ocular symptoms (granulomatous nodules in the conjunctiva, retinal lesions, and periorbital inflammation surrounding dead adult worms) [2]. Nonspecific symptoms, including pruritus, urticaria, fever, pain in bursae and/or joint synovia, enlarged lymph glands, vague abdominal symptoms and fatigue, have also been attributed to *M. perstans* [2]. Headaches, neuropsychiatric manifestations, meningoencephalitis, and hepatitis have also been described. Nonspecific but characteristic laboratory abnormalities include high-level eosinophilia and elevated serum immunoglobulin IgE levels observed in some but not all patients with *M. perstans* infections, likely because of the body’s reaction against the adult worm, rather than against microfilariae [14, 15]. This phenomenon can also be observed in other helminthiasis such as strongyloidiasis and schistosomiasis [16–18]. In our series, all the described features are represented. Moreover, recent observations have suggested that mansonellosis infections can influence the human immune system’s response, which can influence the development of secondary infections, such as malaria [19].

Parasitological diagnosis is based on the detection and identification of sheathless *Mansonella* microfilariae in the skin or blood at any time of day or night. Additionally, the diagnosis is established by identifying the adult worm in tissues. Serologic tests based on crude filarial antigens are useful but do not distinguish between active or past infection and show cross-reactivity among different filarial species and with other nematode infections. Consequently, their usefulness is limited, although a negative result can exclude the possibility of infection. A recent study suggests that ELISA commercial kit can be useful to distinguish between active and past infection [20]. Given the limitations of serology, we do not apply this diagnostic technique and may underestimate the number of cases. Furthermore, have been applied polycarbonate membrane technique or filaria polymerase chain reaction (PCR) serves a special function in the differential detection of filariae in situations where species are co-endemic [1] and the use of PCR could improve the diagnosis of filariae infection.

Among the three types of human mansonellosis, that one caused by *M. perstans* is usually regarded as the most difficult to treat [1, 2]. Our work shows great variability in the treatment of this disease. Therefore, human infection with *M. perstans* raises questions about treatment because of poor responses to standard antifilarial drugs and limited findings from controlled trials. In contrast to conventional anthelmintic treatments, doxycycline has proven to be excellent, effective, and safe in the treatment of *M. perstans* infections [7, 21]. However, the course of treatment over 6 weeks that is necessary for this type of therapy probably makes it impractical for control programs, although it appears to be curative, making it a very desirable therapeutic for travel medicine [7, 21]. *M. perstans* is relatively resistant to standard antifilarial agents, including DEC, ivermectin, albendazole, and mebendazole [4]. The usefulness of doxycycline in the treatment of *M. perstans* varies according to geographic region [7, 22]. An ideal drug treatment for *M. perstans* infections needs to be identified that is effective, fast acting, tolerable and easy to administer. The search for new treatments may include a more meticulous quantitative assessment of the above-mentioned drugs, both alone and in various combinations [2].

Our study had some limitations, which were caused mostly by the retrospective design. First, most patients visited our center because of symptoms or an increased eosinophil count. Thus, the proportion of symptomatic patients is not representative of the general population with *M. perstans* infection. Second, posttreatment follow up was available for only a few patients. Thus, we could not properly describe the response to treatment. Third the conclusions about the effectiviness of the treatment are weak, cause it’s not a clinical trial (randomized with placebo group).

## Conclusions

In summary, a long series of *M. perstans* infections is presented in sub-Saharan immigrants. Mansonellosis should be included in the differential diagnosis with other helminthiasis in patients with pruritus or analytical alterations such as eosinophilia or hyper-IgE presentation. These patients also have a high number of coinfections with other microorganisms, the treatment of which needs to be protocolized.

## Data Availability

The dataset supporting the conclusions of this article is included within the article and its additional file.
